# Rapid Discovery of the Potential Toxic Compounds in *Polygonum multiflorum* by UHPLC/Q-Orbitrap-MS-Based Metabolomics and Correlation Analysis

**DOI:** 10.3389/fphar.2019.00329

**Published:** 2019-04-16

**Authors:** Lifeng Han, Piao Wang, Yulan Wang, Qianyu Zhao, Fang Zheng, Zhiying Dou, Wenzhi Yang, Limin Hu, Caixiang Liu

**Affiliations:** ^1^Tianjin State Key Laboratory of Modern Chinese Medicine, Tianjin Key Laboratory of TCM Chemistry and Analysis, Tianjin University of Traditional Chinese Medicine, Tianjin, China; ^2^CAS Key Laboratory of Magnetic Resonance in Biological Systems, State Key Laboratory of Magnetic Resonance and Atomic and Molecular Physics, National Centre for Magnetic Resonance in Wuhan, Wuhan Institute of Physics and Mathematics, the Chinese Academy of Sciences, Wuhan, China; ^3^Singapore Phenome Centre, Lee Kong Chian School of Medicine, School of Biological Sciences, Nanyang Technological University, Nanyang, Singapore

**Keywords:** *Polygonum multiflorum*, metabolomics, hepatotoxicity, L02 cell, UHPLC/Q-Orbitrap-MS

## Abstract

The dry roots of *Polygonum multiflorum* (PM), involving both the raw and processed materials, are widely used as the traditional Chinese medicine for treating various diseases in China. Hepatotoxicity has been occasionally reported in patients who consume PM. Unfortunately, no definite criteria are currently available regarding the processing technology of PM for reduction the toxicity. In this work, we aimed to investigate the variations of PM metabolite profiles induced by different processing technologies by UHPLC/Q-Orbitrap-MS and multivariate statistical analysis, and to discover the potential toxic compounds by correlating the cytotoxicity of L02 cell with the contents of metabolites in raw and processed PM samples. We could identify two potential toxic compounds, emodin-8-*O*-glucoside and torachrysone-*O*-hexose, which could be selected as the toxic markers to evaluate different processing methods. The results indicated all processed PM samples could decrease the cytotoxicity on L02 cell. The best processing technology for PM process was to steam PM in black soybean decoction (BD-PM) for 24 h.

## Introduction

The root of *Polygonum multiflorum* (PM) serves as a popular traditional Chinese medicine (TCM) frequently in medicines and prescriptions for treating many diseases. PM has multiple medicinal activities and biological effects, such as anti-aging, immunomodulation, anti-hyperlipidemia, hepatic protection, and anti-inflammation. Phytochemical investigations have reported various classes of active compounds like stilbenes, anthraquinones, phenolic acids, and flavonoids (Chen et al., 2001; Jiang et al., 2006; Yi et al., 2007; Xu et al., 2009; Wen et al., 2012; Li et al., 2013; Qiu et al., 2013; Sun et al., 2013; Thiruvengadam et al., 2014; Lin et al., 2015; Liu et al., 2015; Chang et al., 2016; Wang et al., 2017). However, PM was also reported to have severe toxic effects, especially the hepatotoxicity (Wu et al., 2012; Dong et al., 2015). Collections of studies have been conducted to discover the hepatotoxic compounds (But et al., 1996; Wong et al., 2005; Cárdenas et al., 2006; Jung et al., 2011; Li et al., 2015).

Processing of herbal medicine has a long history in TCM. The processing procedures typically include steaming, baking, soaking, or other methods, to enhance efficacy and/or reduce toxicity (Yu et al., 2017). It was recommended to steam PM with black soybean decoction (BD) by the Chinese Pharmacopeia (2015 edition). Some other processing technologies, such as steaming PM with water (W), yellow rice wine (YRW), or black soybean decoction, and yellow rice wine (BY), have been documented (Cui et al., 2016). Different processing procedures could result in differentiated chemical components that are extracted from PM (Li et al., 2018). It ultimately affects the efficacy and/or toxicity of the processed PM (P-PM). Furthermore, geographical origin and harvesting time could influence the metabolites in PM, which might result in different pharmacological effects (Li et al., 2007; Chen et al., 2008).

Recent developments of the analytical techniques, especially ultra-high performance liquid chromatography (UHPLC) by applying sub-2-μm particles, have shown significant improvements in the chromatographic resolution, selectivity, sensitivity, and reproducibility (Liu et al., 2011; Zhao et al., 2012). Q-Orbitrap-MS is a standalone orbitrap HRMS platform with diverse scan modes suitable for high-throughput qualitative and quantitative analyses (Michalski et al., 2011). Metabolomics has been principally used to evaluate metabolite changes in samples obtained from different pathological or physiological conditions (Li Y. et al., 2017). Thousands of metabolites could be analyzed simultaneously for biomarkers discovery by UHPLC-HRMS-based metabolomics, in phenotyping and diagnostic analyses in clinical treatments and in plant materials (Fernie and Schauer, 2009; Roux et al., 2012; Armitage et al., 2013; Glauser et al., 2013).

In this work, we established a reliable untargeted metabolomics method for comparing the metabolite variations among raw PM (R-PM) and different processed PM (P-PM) samples by UHPLC/Q-Orbitrap-MS. Toxicity was evaluated on L02 cell. Multivariate statistical analysis combined with correlation analysis were utilized to discover the potential hepatotoxic compounds. Our current work demonstrated that metabolomics is a useful vehicle in monitoring the overall metabolite changes and evaluating the processing technology of TCM.

## Materials and Methods

### Plant Materials

Raw materials of PM were purchased from Anguo Qi An Pharmaceutical Co., Ltd., (Hebei, China) and authenticated by professor Lijuan Zhang from Tianjin University of Traditional Chinese Medicine. Voucher specimen was deposited in our laboratory (Wang et al., 2017).

A total of 900 g of PM was divided into four groups and soaked with pure water (100:120, *w*_R-PM_*/w*_water_), black soybean juice (100:10, *w*_R-PM_*/w*_BD_), yellow rice wine (100:12, *w*_R-PM_*/w*_Y RW_), black soybean plus yellow rice wine (100:10:25, *w*_R-PM_*/w*_BD_*/w*_Y RW_) for 12 h, respectively. The soaked PM samples afterward were steamed and sampled at 6, 12, 18, 24, 32, and 36 h, respectively. Different P-PM samples were dried in an oven under 45°C for 24 h. The black soybean juice was in-house prepared by separately decocting 0.18 kg of black soybean with 1.44 and 1.08 L of pure water for 4 and 3 h, respectively. The filtered decoction was combined and concentrated to approximately 0.45 L.

### Chemicals and Reagents

Acetonitrile of HPLC grade was purchased from Thermo Fisher Scientific (Pittsburgh, PA, United States) and acetic acid (≥99.7%) was obtained from Sigma-Aldrich Corporation (Saint Louis, MO, United States). Deionized water was obtained using a Millipore Milli-Q water system (Bedford, MA, United States). Other reagents were of analytical grade.

Reference standards, including gallic acid, physcion, emodin, emodin-8-*O*-glucoside, apigenin, oxyresveratrol, protocatechuate, and 2,3,5,4’-tetrahydroxystilbene-2-*O*-*β*-D-glucoside (TSG), were purchased from National Institutes for Food and Drug Control (Beijing, China). D-fructose, malic acid, gluconic acid, sucrose, D-tryptophan, linoleic acid, catechin, and daidzein (>98%) were purchased from Shanghai Yuanye Biological Technology Co., Ltd., (Shanghai, China). The manufacturer of yellow rice wine was Shaoxing No. 3 wine factory in Shaoxing city of Zhejiang Province, and the batch number was 16112506.

### Sample Preparations

An aliquot of 1 g of crushed R-PM and different P-PM samples were accurately weighed and extracted with 8 mL of 60% ethanol (EtOH) ultrasonically for 90 min (avoid sunlight). The obtained solutions were filtered and centrifuged at 13,200 × *g* for 10 min. The resultant supernatants were diluted with 60% EtOH to reach a concentration of 6.25 mg ⋅ mL^-1^. Six replicates were prepared for each processing method and each time-point, and a total of 150 samples were obtained.

We used RANK formula to inject all the 150 samples, the first sample in the random sequence was chosen as QC sample, and BD-PM-36h_1 was chosen finally.

### UHPLC/Q-Orbitrap-MS Analysis

Untargeted metabolomics was accomplished with Thermo Fisher Scientific U3000 UHPLC equipped with Q Exactive^TM^ Q-Orbitrap MS through an HESI source. Chromatographic separation was achieved on a Waters BEH C_18_ column (2.1 × 100 mm, 1.7 μm). Temperature for the column oven and automatic sampler was set at 35 and 4°C, respectively. The mobile phase consisted of 0.1% acetic acid in water (A) and acetonitrile (B) with a flow rate of 0.4 mL ⋅ min^-1^, running in accordance with a gradient ranging from 3 to 97% of B within 20 min. The injection volume was 2 μL.

High-accuracy MS data were acquired in the negative mode by Full MS/dd-MS^2^. The HESI source parameters were as follows: spray voltage, 3.50 kV; sheath gas rate (N_2_), 35 L ⋅ h^-1^; auxiliary gas rate (N_2_), 10 L ⋅ h^-1^; capillary temperature, 320°C; and auxiliary gas heater temperature, 350°C. The Orbitrap mass analyzer scanned over a range of *m/z* 100 to 1500. All the data were recorded and processed by Thermo Scientific Xcalibur 3.0 software (Thermo Fisher Scientific).

### QC Samples and Run Order

In our study, the solvent 60% EtOH was selected as the blank control for background subtraction. A Quality Control (QC) sample was utilized to guarantee the stability and precision of measurement and the samples were injected for analysis at random.

### Data Processing

Processing of the metabolomics data was performed by SIEVE 2.2 (Thermo Fisher Scientific) for background subtraction and components extraction. The obtained peak list was further processed by principal component analysis (PCA) and Student’s *t*-test. SIMCA-P 14.1 (Umetrics AB, Umeå, Sweden) was used for data transformation for orthogonal partial least squares discriminant analysis (OPLS-DA). Metabolites satisfying both VIP >1.0 and *p* < 0.05 were chosen concurrently as the markers for differentiating P-PM from R-PM. Metlin database^1^, *m/z* cloud^2^, HMDB^3^ as well as reference compounds comparison, were utilized for putative identification of the significantly differential metabolites. Additionally, multi experiment viewer (MEV) software was used for the generation of heatmap. GraphPad Prism 6 was used for producing box plots and line graphs. Fragmentation trees were formed in analog by Sirius (Version 4.0).

### Cell Culture

Human hepatocyte cell line L02 was purchased from China Cell Culture Center (Shanghai, China). L02 cells were maintained in DMEM Medium (Hyclone, United States), containing 10% fetal bovine serum (Gibco, United States) and Penicillin-Streptomycin Solution (100×, Solabio, China) at 37°C, under the atmosphere with 95% air and 5% CO_2_. 0.25% trypsin (Amresco, United States) was used to passage cells at 80∼90% confluence. The medium was changed every 3 days.

### Flow Cytometry Test

For cell death identification, L02 cells were seeded in 6-well culture plates and treated with different extracts of R-PM and P-PM (same comparable doses of herbal medicines about 40 μg ⋅ mL^-1^). The cells were harvested after 24 h followed by washing three times in ice-cold PBS, and then analyzed with an annexin V-FITC apoptosis detection kit (BD, United States) using a flow cytometer (Becton-Dickinson Accun C6, San Jose, CA, United States).

## Results

### Method Validation

To ensure the reliability and reproducibility of analytical method, 26 injections of QC sample were implemented. The retention time and peak area of 10 peaks were selected for consistent evaluations. As showed in Table 1, the RSD values of retention time and peak area of the 10 peaks were all less than 5%, suggesting a good precision and stability of methodology.

**Table 1:** Precision of ten peaks selected from QC sample.

Peak No.	1	2	3	4	5	6	7	8	9	10
	(Comp. 11)	(Comp. 37)	(Comp. 49)	(Comp. 63)	(Comp. 87)	(Comp. 110)	(Comp. 120)	(Comp. 131)	(Comp. 134)	(Comp. 135)
Rt (min)	0.63	1.30	3.22	4.95	7.33	9.90	11.82	17.23	18.01	19.04
m/z	179.0551	169.0140	289.0716	405.1193	431.0977	283.0610	269.0452	227.2015	279.2326	255.2329
Rt (RSD%)	0.00	1.36	0.51	0.32	0.18	0.17	0.15	0.09	0.09	0.10
Peak area (RSD%)	4.17	4.81	2.03	4.85	4.61	4.91	4.86	4.64	4.74	4.73

*Comp. 11: D-Fructose; Comp. 37: Gallic acid; Comp. 49: Catechin; Comp. 63: TSG; Comp. 87: Emodin-8-O-glucoside; Comp. 110: Isomer of physcion; Comp. 120: Emodin; Comp. 131: Tetradecanoic acid; Comp. 134: Linoleic acid; Comp. 135: n-Hexadecanoic acid.*

### Identification of Metabolites From R-PM and P-PM

**FIGURE 1:**
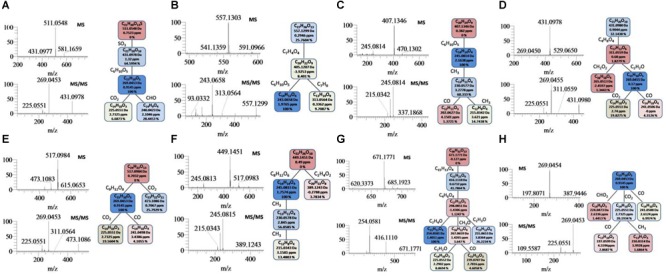
Examples for the identification of several metabolites. **(A)**: Emodin-8-*O*-*B*-D-hexose-sulfate; **(B)**: tetrahydroxystilbene-(9-(galloyl)-glucoside; **(C)**: torachrysone-*O*-hexose; **(D)**: emodin-8-*O*-glucoside; **(E)**: emodin-8-*O*-(6’-0-carboxyacetyl)-/?-D-glucoside; **(F)**: torachrysone-*O*-(acetyl)-hexose; **(G)**: emodin (10-10’)-emodin monosaccharide glucoside; (H): ernodin.

After optimizing SIEVE parameters, 136 metabolic features including *m/z-*value and retention time were extracted. As shown in Supplementary Table S1, 69 features were tentatively identified according to characteristic diagnostic fragment ions, and 16 thereof them were identified by comparison with reference standards. The typical total ion chromatogram (TIC) was shown in Supplementary Figure S1 and identification of eight representative metabolites based on high-accuracy MS and MS/MS spectra was exhibited in Figure 1. Parent ions of [M-H]^-^ at *m/z* 511.0548 (C_21_H_19_O_13_S) were observed in the MS^1^ spectrum. The diagnostic fragment ions at *m/z* 431.0978 (C_21_H_19_O_10_) and *m/z* 269.0453 (C_15_H_9_O_5_) were corresponding to the loss of a 80 Da (-SO_3_ group) and 80+162 Da (-SO_3_ + hexose group), respectively. The fragment ions such as *m/z* 240.0423, 225.0551, and 269.0453, could indicate the presence of an emodin skeleton. Ultimately, this metabolite was tentatively identified as emodin-hexose-sulfate (Krenn et al., 2003; Comp. 82).

Comp. 74 gave [M-H]^-^ ions at *m/z* 557.1303 (C_27_H_25_O_13_) in the negative MS spectrum Figure 1B. The characteristic product ions at *m/z* 405.1207 (C_20_H_21_O_9_) could indicate the elimination of a galloyl group (152 Da). The diagnostic ions at *m/z* 243.0658 could be attributed to successive losses of a galloyl and a hexose group. Two key fragments, consistent with deprotonated gallic acid, at *m/z* 169.0133 (C_7_H_5_O_5_) and *m/z* 125.0231 (C_6_H_5_O_3_) were observed. By comparing with literature (Wang et al., 2015), Comp. 74 was tentatively identified as tetrahydroxystilbene-*O*-(galloyl)-glucopyranoside (Wang et al., 2005). Similar parent and product ions were observed in the MS^1^ and MS^2^ spectra of Comp. 70 and 73, based on which we characterized them as the isomers of Comp. 74 (Supplementary Table S1).

According to the similar methods afore-mentioned, Comp. 83 (Figure 1C), 87 (Figure 1D), 90 (Figure 1E), 91 (Figure 1F), 112 (Figure 1G), and 120 (Figure 1H) were identified or tentative identified as torachrysone-*O*-hexose, emodin-8-*O*-glucoside, emodin-8-*O*-(6′-*O*-carboxyacetyl)-*β*-D-glucoside, torachrysone-*O*-(acetyl)-hexose, emodin (10–10′)-emodin monosaccharide glucoside and emodin, respectively (Supplementary Table S1).

**FIGURE 2:**
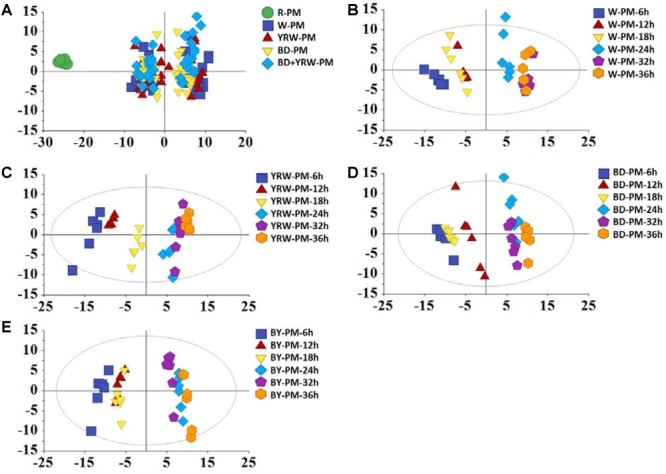
PC A scores plots of R-PM and four kinds of P-PM at different processing time for **(A)** all samples, **(B)** water-processed samples (W-PM), **(C)** yellow rice wine-processed samples (YRW-PM), **(D)** black soybean juice-processed samples (BD-PM), and **(E)** black soybean plus yellow rice wine-processed samples (BY-PM) at all time-points.

Score plot of PCA showed clear separations between R-PM and P-PM (Figure 2A). It was evident that, the four groups of P-PM samples all displayed two clusters related to the processing time (6–18 and 24–36 h) (Figure 2B–E).

**FIGURE 3:**
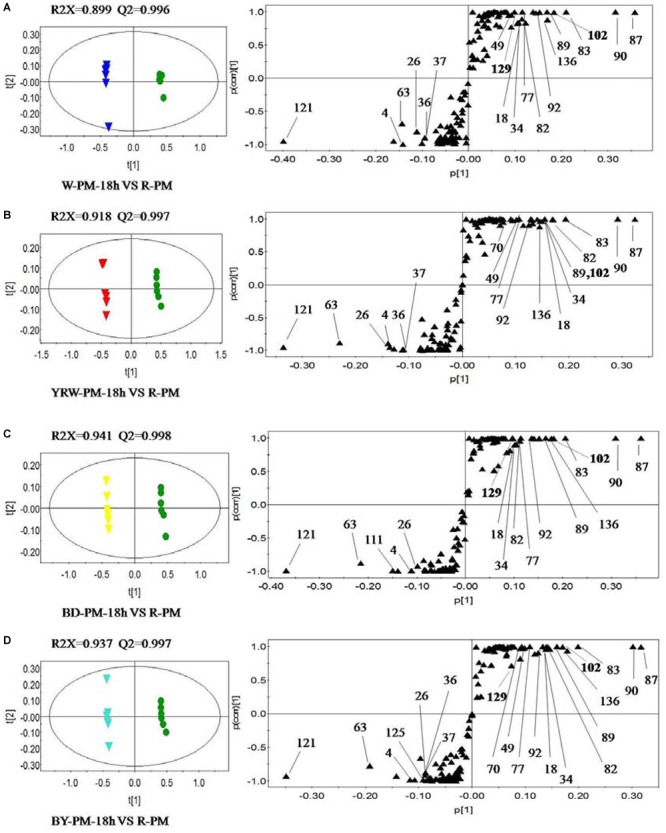
OPLS-DA scores plots (left) and S-plots (right) showing the metabolic differences between four kinds of P-PM and R-PM for **(A)** water-PM (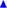 vs. R-PM 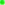), **(B)** YRM-PM (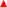) vs. R-PM (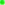), **(C)** BD-PM (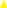) vs. R-PM (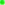), and **(D)** BY-PM (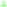) vs. R-PM (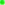) at l8 h (The numbers of metabolites in S-plot were identical with those in Supplementary Table SI).

According to the results of PCA, in the next step, we selected two representative time-points (18 and 36 h) for further comparative analysis. OPLS-DA was utilized to classify different groups and further probe differential components. In order to identify the significantly altering metabolites related to the processing methods, the VIP value (VIP >1.0) from multivariate data analysis and *p-*value (*p* < 0.05) of *t*-test between the R-PM and 4 groups of P-PM were chosen as the criteria for markers discovery. Figure 3 showed, compared to R-PM, all P-PM samples with a processing time 18 h contained higher levels of kaempferol isomer (Comp. 121), gluconic acid (Comp. 4), 2,3,5,4′-tetrahydroxystilbene-2-*O*-*β*-D-glucoside (TSG, Comp. 63), and malic acid (Comp. 26). However, the contents of emodin-8-*O*-glucoside (Comp. 87), emodin-8-*O*-(6′-*O*-carboxyacetyl)-*β*-D-glucoside (Comp. 90), aurantio-obtusin (Comp. 102), torachrysone-*O*-hexose (Comp. 83), aloe-emodin-8-*O*-(6′-*O*-acetyl)-glucoside (Comp. 89), apigenin (Comp. 92), emodin-8-*O*-*β*-D-hexose-sulfate (Comp. 82), apigenin-7-*O*-glucoside (Comp. 77), isomer of sucrose (Comp. 34), sucrose (Comp. 18) and 9-octadecadienoic acid (Comp. 136), remarkably decreased in all P-PM samples obtained at 18 h. In addition, except BD-PM, other three kinds of P-PM samples all involved higher levels of gallic acid (Comp. 37) and its isomer (Comp. 36), together with lower levels of catechin (Comp. 49), in contrast to R-PM (Figure 3). Furthermore, compared with R-PM, YRW-PM exhibited lower levels of tetrahydroxystilbene-*O*-(galloyl)-glucopyranoside (Comp. 70) (Figure 3B); BD-PM had higher levels of malonyl-substitution of dianthrone glycoside (Comp. 111) (Figure 3C), whilst BY-PM contained higher level of an unknown (Comp. 125) and lower level of tetrahydroxystilbene-*O*-(galloyl)-glucopyranoside (Comp. 70) (Figure 3D; Table 2).

**Table 2:** Identification of significant changed metabolites from four kinds of P-PM compared with R-PM for 18 h^a^ (the first) and 36 h^b^ (the second).

Comp	Compounds	W vs. R	YRW vs. R	BD vs. R	BY vs. R
2	377.0851	—^a^↓^b^	↓↓	—↓	↓↓
4	Gluconic acid	↑↑	↑↑	↑↑	↑↑
7	215.0326	↑↑	↑↑	—↑	↑↑
16	379.0820	—↓	—↓	—↓	—↓
17	404.1042	↓↓	↓↓	↓↓	—↓
18	Sucrose	↓↓	↓↓	↓↓	↓↓
26	Malic acid	↑↑	↑↑	↑↑	↑↑
30	191.0188	——	—↑	——	↑↑
34	Isomer of sucrose	↓↓	↓↓	↓↓	↓↓
35	377.0851	—↓	↓↓	—↓	↓↓
36	Isomer of gallic acid	↑↑	↑↑	—↑	↑↑
37	Gallic acid	↑↑	↑↑	—↑	↑↑
49	Catechin	↓↓	↓↓	—↓	↓↓
50	121.0282	——	—↑	——	—↑
63	TSG	↑↑	↑↑	↑—	↑↑
70	Tetrahydroxystilbene-O-(galloyl)-glucopyranoside	—↓	↓↓	—↓	↓—
77	Apigenin-7-O-glucoside	↓↓	↓↓	↓↓	↓↓
82	Emodin-8-O-β-D-hexose-sulfate	↓↓	↓↓	↓↓	↓↓
83	Torachrysone-O-hexose	↓↓	↓↓	↓↓	↓↓
86	408.1379	↓—	↓—	↓—	↓—
87	Emodin-8-O-glucoside	↓↓	↓↓	↓↓	↓↓
89	Aloe-emodin-8-O-(6’-O-acetyl)-glucoside	↓↓	↓↓	↓↓	↓↓
90	Emodin-8-O-(6’-O-carboxyacetyl)-β-D-glucoside	↓↓	↓↓	↓↓	↓↓
92	Apigenin	↓↓	↓↓	↓↓	↓↓
96	481.0906	↓↓	↓↓	↓↓	↓↓
102	Aurantio-obtusin	↓↓	↓↓	↓↓	↓↓
111	Malonyl-substitution of dianthrone glycoside	—↑	—↑	↑—	—↑
118	431.8842	↑↑	↑↑	↑↑	↑↑
121	Isomer of kaempferol	↑↑	↑↑	↑↑	↑↑
125	233.1540	——	——	——	↑—
129	Emodin (10/10’) physcion dianthrone glycoside	↓—	——	↓—	↓—
136	9-Octadecadienoic acid (9Z)	↓↑	↓—	↓↑	↓—

*↑: means the relative contents increased significantly; ↓: means the relative contents decreased significantly; —: means the relative contents didn’t change significantly.*

OPLS-DA between R-PM and different P-PM groups at 36 h (Supplementary Figure S2) was conducted as well. In detail, comparing to R-PM, P-PM samples all contained higher levels of kaempferol isomer (Comp. 121), gluconic acid (Comp. 4), gallic acid (Comp. 37), isomer of gallic acid (Comp. 36), and malic acid (Comp. 26) and lower levels of emodin-8-*O*-glucoside (Comp. 87), torachrysone-*O*-hexose (Comp. 83), aloe-emodin-8-*O*-(6′-*O*-acetyl)-glucoside (Comp. 89), apigenin (Comp. 92), catechin (Comp. 49), emodin-8-*O*-*β*-D-hexose-sulfate (Comp. 82), apigenin-7-*O*-glucoside (Comp. 77), sucrose (Comp. 18) and its isomer (Comp. 34), and emodin-8-*O*-(6′-*O*-carboxyacetyl)-*β*-D-glucoside (Comp. 90) (Supplementary Figures S2, S3). Additionally, compared with R-PM, W-PM, and BD-PM groups had higher levels of 9-octadecadienoic acid (9Z) (Comp. 136), while W-PM, YRW-PM, and BY-PM, had more abundant of TSG (Comp. 63). Except for BY-PM, the other three kinds of P-PM all had tetrahydroxystilbene-*O*-(galloyl)-glucopyranoside (Comp. 70) at a lower content (Supplementary Figure S2). Very interestingly, malonyl-substitution of dianthrone glycoside (Comp. 111) was enriched after processing except BD-PM (Supplementary Figure S2). Other significantly changed metabolites failing to be identified were listed with their *m/z*-values in Table 2.

**FIGURE 4:**
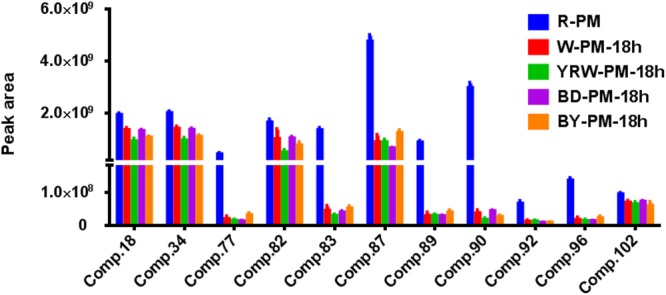
The abundance map of the preferred markers from OPLS-DA analysis among R-PM and P-PM at 18 h.

Based on the data shown in Table 2, we could conclude that, processing was able to increase the contents of four common components (Comp. 4, 26, 118, and 121) and simultaneously decrease the levels of eleven metabolites (Comp. 18, 34, 77, 82, 83, 87, 89, 90, 92, 96, and 102). Figure 4 showed the abundance map of the eleven decreased markers in P-PM at 18 h compared to R-PM. The results were coincident with that of OPLS-DA and S-plot.

Additionally, eight compounds (Comp. 2, 16, 17, 35, 49, 70, 86, and 129) showed a decreasing trend from 18 to 36 h post processing. According to the processing theory of TCM, the purpose of processing is to increase effectiveness and decrease the toxicity, therefore, we could primarily speculate the toxic compounds of PM should be among these 19 metabolites that displayed the decreasing tendency after processing.

### Correlation Analysis Between the Toxicity Assay and Metabolites Variation

**FIGURE 5:**
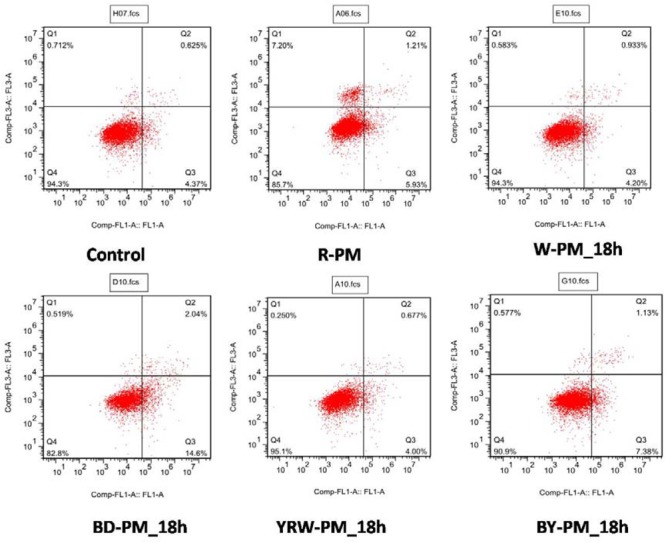
Flow cytometry results of R-PM and different P-PM at 18 h (Ql: death cells; Q2˜Q3: apoptosis cells; Q4: survival cells).

Untargeted metabolomic studies enabled the unveiling of a series of potential toxic compounds, however, the toxicity of these compounds should be further confirmed. All the extracts of R-PM and P-PM samples were evaluated for the apoptosis activities on L02 cell and viewed by a flow cytometry method. Here, Q1 denoted the percentage of dead cells, and Q2–Q3 indicated the apoptosis cells, while Q4 represented the survival cells. Evidently, all the P-PM samples exhibited toxicity lower than R-PM. Figure 5 illustrated the typical results of flow cytometry, which indicated R-PM had more dead cells (Q1) than all four kinds of P-PM samples at 18 h on L02 cell. These results could support the processing theory of TCM.

**FIGURE 6:**
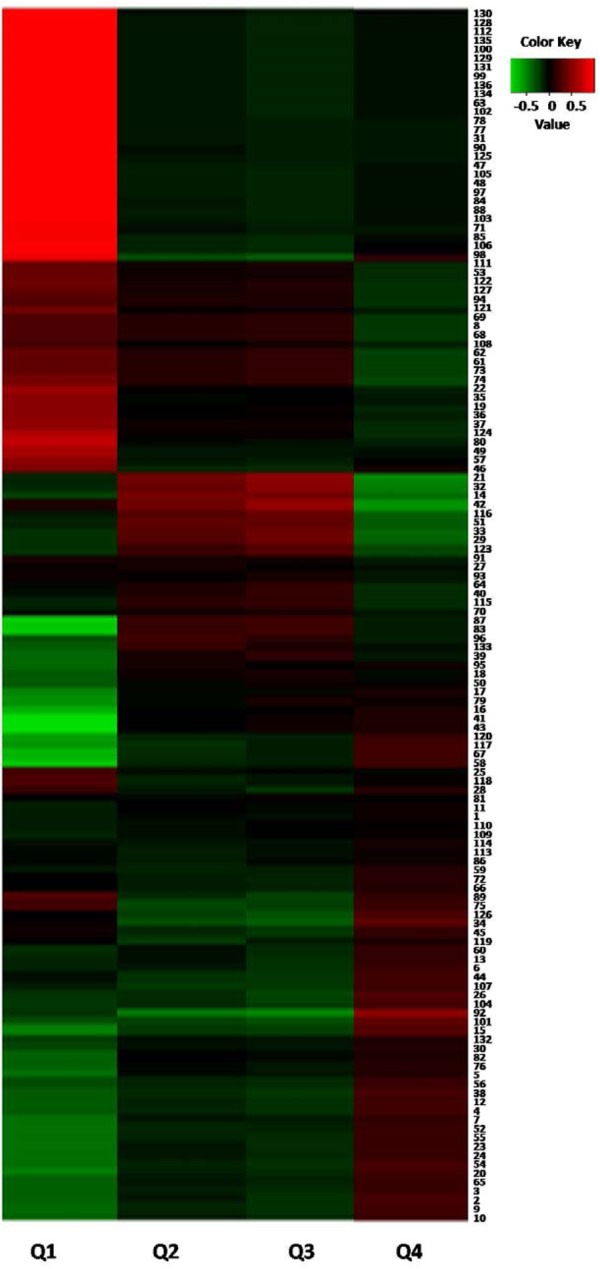
Correlation analysis between cytotoxicity and different metabolites from P-PM. (The numbers in the right part were identical with that of Supplementary Table SI).

In order to verify the metabolomic results and discover the toxic compounds in PM, correlation analysis between flow cytometry results and metabolites variations in different PM samples were further performed by an in-house R program (Xu et al., 2012). Features annotated as kaempferol (Comp. 94), *m/z* 269.0121 (Comp. 69), *m/z* 135.0285 (Comp. 8), *m/z* 441.0967 (Comp. 68), oxyresveratrol (Comp. 62), *m/z* 439.1236 (Comp. 61), tetrahydroxystilbene-*O*-(galloyl)-glucopyranoside (Comp. 73), isomer of tetrahydroxystilbene-*O*-(galloyl)-glucopyranoside (Comp. 74), protocatechuate (Comp. 42), *m/z* 128.0341 (Comp. 33), *m/z* 111.0074 (Comp. 29), aloe emodin dianthrone (Yang et al., 2005) (Comp. 123), *m/z* 473.2175 (Comp. 115), emodin-8-*O*-glucoside (Comp. 87), torachrysone-*O*-hexose (Comp. 83), *m/z* 481.0906 (Comp. 96), and dethiobiotin (Comp. 39) were negatively correlated with survival (Q4) and positively correlated with apoptosis (Q2 and Q3), which could confirm that these compounds were the toxic compounds in PM (Figure 6).

### Identification of the Toxic Components

We have identified 17 toxic compounds by correlation analysis. Combination of the 19 potential toxic compounds obtained in OPLS-DA and S-plot analysis and 17 compounds from correlation analysis, three common compounds were discerned, which showed decreasing tendency. They were torachrysone-*O*-hexose (Comp. 83), emodin-8-*O*-glucoside (Comp. 87) and *m/z* 481.0906 (Comp. 96). Unfortunately, the last compound failed to be characterized.

## Discussion

Comp. 96 is an unknown compound, further experiments will be performed to fully establish its structure in our future continuous investigation. Two compounds, torachrysone-*O*-hexose (Comp. 83) and emodin-8-*O*-glucoside (Comp. 87) could be identified as the toxic compounds, which have been previously reported (Lv et al., 2015; Lin et al., 2017; Yang et al., 2018). For example, several anthraquinones including emodin-8-*O*-glucoside (Comp. 87) and naphthols including torachrysone-*O*-hexose (Comp. 83) were reported as the toxic components against the zebrafish embryos (Yang et al., 2018).

In addition to torachrysone-*O*-hexose (Comp. 83) and emodin-8-*O*-glucoside (Comp. 87), several other compounds have been previously pinpointed as the toxic compounds. For example, tetrahydroxystilbene-*O*-(galloyl)-hex (Comp. 73 and 74) has been suggested to be toxic (Lin et al., 2017). Several literatures focused on emodin (Comp. 120) as the hepatotoxic compound (Lv et al., 2015; Yang et al., 2018), however, in our study, its contents were increased due to the deglycosyl reactions of emodin-glucosides. The flow cytometry results showed that emodin was negatively correlated with apoptosis (Q2 and Q3). Therefore, the hepatotoxicity of emodin should need further verification.

**FIGURE 7:**
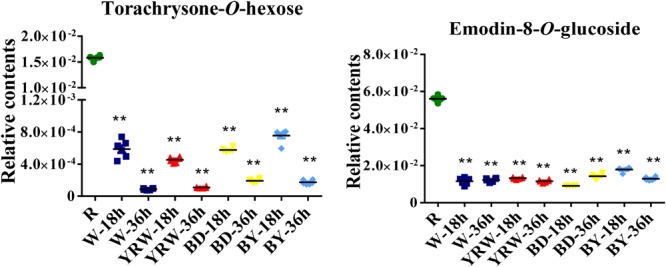
Statistic analysis of two metabolites of R-PM and different P-PM (BY-PM: BD+YRW-PM; ^∗∗^*p* < 0.01, compare with R-PM).

Subsequently, we measured the levels of torachrysone-*O*-hexose (Comp. 83) and emodin-8-*O*-glucoside (Comp. 87) at 18 and 36 h post processing. We found that their contents were decreased significantly in all four kinds of P-PM, compared with R-PM (Figure 7). In addition, the content of torachrysone-*O*-hexose at 36 h time-point was much lower than that of 18 h time-point (Figure 7). Combining with metabolomics analysis results, these two compounds (torachrysone-*O*-hexose and emodin-8-*O*-glucoside) are the potential toxic compounds in PM. The two compounds could also be used as toxic index for further evaluation of different processing technologies.

**FIGURE 8:**
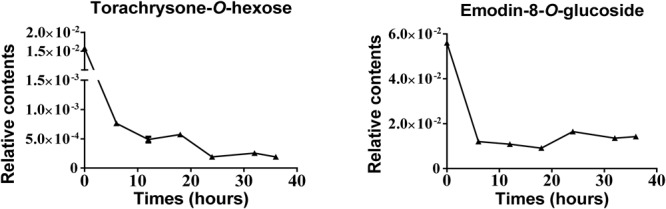
Line graphs of two metabolites at six processing times in BD-PM.

Further analysis was performed by focusing on BD-PM samples, since this processing technology is the most commonly used and has been recommended by Chinese Pharmacopeia. According to the line chart in Figure 8, the two potential toxic compounds (Comp. 83 and 87) were decreased significantly, and the best processing time seemed to be between 24 to 36 h because these two compounds were at almost the lowest concentrations at this time range. Since no further significant reduction in the levels of the two compounds after 24 h of post processing was observed, by considering of the economic value of short processing time and the meantime taking the recommendation of Chinese Pharmacopeia, we could deduce that the best technology for BD-PM was steamed for 24 h. Our results also showed that all the four methods could decrease the cell toxicity *in vitro*. From the OPLS-DA and S-plot analysis, the characteristic compounds in four technologies were similar. Therefore, the scientific evidence for raw PM steamed with BD (black soybean) decoction as recommended by Chinese Pharmacopeia needs to be further investigated.

A recent report with respect to the herb induced liver injury (HILI) indicated that the most frequently reported hepatotoxic herb was PM, which occupied 32.3% (108 cases) of the known HILI causative herbs (Byeona et al., 2019). In fact, although PM-induced hepatotoxity has been reported widely, its idiosyncratic liver injury (IDILI) was generally accepted based on integrated evidence chain-based identification of Chinese herbal medicine (Wang et al., 2015; Liu et al., 2018). However, discovery of idiosyncratic hepatotoxic components has been a formidable challenge.

The mechanism of IDILI was very complex, and inflammation response played a critical role. Therefore, the LPS model which based on the inflammatory stress hypothesis has been employed to evaluate IDILI extensively in toxicological experiment (Gao et al., 2016; Li et al., 2016). Utilizing the co-treated LPS model, PM was confirmed to induce acute liver injury when administrated the clinical equivalent dose on rats (Li et al., 2015). However, the idiosyncratic hepatotoxic components in PM remained unclear. Recently, 2,3,5,4′-tetrahydroxy *cis*-stilbene-2-*O*-*β*-glucoside (*cis*-TSG) was reported to be the idiosyncratic hepatotoxic compound (Li C.Y. et al., 2017; Meng et al., 2017). However, this *cis*-compound can usually be generated from its *trans*- form by exposure to ultraviolet light or sunlight. Several studies have demonstrated anthraquinones, such as emodin and emodin-8-*O*-glucoside, are the major hepatotoxins in PM (Lin et al., 2015; Lv et al., 2015; Ma et al., 2015; Yang et al., 2018). Anthraquinones herb toxicity has also been reported to associate with highly reactive anthrones in the colon and induce hepatotoxicity (Westendorf, 1993). Furthermore, some reports indicated that the ethyl acetate (EA) extract of PM was the main components related with IDILI and further analytical research confirmed that the EA extract mainly contained stilbenes and emodin-8-*O*-*β*-D-glucopyranoside (Li et al., 2016). Therefore, further researches on the evaluation of torachrysone-*O*-hexose and emodin-8-*O*-glucoside on LPS induced IDILI model need to be done.

## Conclusion

Although there were several reports on the hepatotoxic compounds of PM, they were either evaluated by pure compounds or targeted analysis of different PM samples. Both of them are in lack of the integration of chemical constituents and toxic activities. In this study, we elaborated a metabolomics approach to analyze the variation of chemical constituents during the processing of PM, and integrated toxic activity evaluations. Both OPLS-DA analysis of the metabolites variations in post processing of PM and the correlation analysis between flow cytometry test and different metabolites in post processed PM enabled the identification of two toxic compounds. They were torachrysone-*O*-hexose (Comp. 83) and emodin-8-*O*-glucoside (Comp. 87) which both decreased significantly after processing. These two toxic compounds could be used as the potential toxic markers of PM. Further investigation of these two toxic compounds in the post processed samples suggested the best processing time was 24 h. Our experiments successfully validated two toxic compounds in PM, and provided enriched information for Chinese Pharmacopeia on the processing time of PM.

## Author Contributions

LfH and PW performed the experiments and wrote the manuscript. QZ and FZ analyzed the data. LmH, CL, and YW guided the experiments. ZD, CL, WY, and YW revised the manuscript. LmH acquired funding for the research. All the authors read and approved the final manuscript.

## Conflict of Interest Statement

The authors declare that the research was conducted in the absence of any commercial or financial relationships that could be construed as a potential conflict of interest. The handling Editor and reviewer Jb-W declared their involvement as co-editors in the Research Topic, and confirm the absence of any other collaboration.

## Footnotes

1 http://metlin.scripps.edu/index.php

2 https://www.mzcloud.org/

3 http://www.hmdb.ca/
