# Supplementary material for: Rapid Discovery of the Potential Toxic Compounds in Polygonum multiflorum by UHPLC/Q-Orbitrap-MS-Based Metabolomics and Correlation Analysis
Source: Front Pharmacol. 2019 Apr 16;10:329. doi: 10.3389/fphar.2019.00329 (PMC6477936; doi:10.3389/fphar.2019.00329)
Supplement: Supplementary file 1 [file Data_Sheet_1.doc]

**Untargeted metabolomic analysis of** **different processing methods on *Polygonum multiflorum* based on UHPLC-Q-Orbitrap-MS**

**Lifeng Han^1,2†^, Piao Wang^1†^, Yulan Wang^2,3*^, Qianyu Zhao^1^, Fang Zheng^1^, Zhiying Dou^1^,Wenzhi Yang^1^, Limin Hu^1^ and Caixiang Liu^2*^**

*^1^ Tianjin State Key Laboratory of Modern Chinese Medicine, Tianjin Key Laboratory of TCM Chemistry and Analysis, Tianjin university of Traditional Chinese Medicine, 312 Anshanxi Road, Nankai District, Tianjin 300193, China*

*^2^ CAS Key Laboratory of Magnetic Resonance in Biological Systems, State Key Laboratory of Magnetic Resonance and Atomic and Molecular Physics, National Centre for Magnetic Resonance in Wuhan, Wuhan Institute of Physics and Mathematics, the Chinese Academy of Sciences, Wuhan 430071, China*

*^3^ Singapore Phenome Centre, Lee Kong Chian School of Medicine, School of Biological Sciences, Nanyang Technological University, 636921, Singapore*

**TABLE S1** Identification of features in raw and processed *polygonum multiflorum*.

| **Comp.** | **Rt (min)** | ***m/z* (Neg)** | **Formula** | ***δ* ppm** | **MS/MS (*m/z*)** | **Identification** |
| --- | --- | --- | --- | --- | --- | --- |
| **1** | 0.52 | 201.0247 | - | - | 59.0125, 141.8669, 164.8350, 159.8778, 166.8322 | - |
| **2** | 0.62 | 377.0851 | - | - | 341.1083, 215.0323, 89.0229, 59.0125, 179.0550, 113.0228 | - |
| **3** | 0.62 | 537.1667 | - | - |  | - |
| **4** | 0.62 | 195.0505 | C_6_H_12_O**_7_** | 2.70 | 75.0074, 129.0180, 87.0074, 59.0125, 99.0074, 71.0125, 159.0285 | Gluconic acid***** |
| **5** | 0.62 | 294.0830 | - | - |  | - |
| **6** | 0.63 | 719.2011 | - | - |  | - |
| **7** | 0.63 | 215.0326 | - | - | 71.0125, 89.0230, 59.0124, 101.0231, 113.0230, 193.0687, 179.0559, 163.0674 | - |
| **8** | 0.63 | 135.0285 | - | - | 116.0061, 117.0069, 71.0126, 75.0076, 72.9919 | - |
| **9** | 0.63 | 507.1559 | - | - | 165.0394, 75.0074, 129.0181, 147.0288 | - |
| **10** | 0.63 | 267.0725 | - | - |  | - |
| **11** | 0.63 | 179.0551 | C_6_H_12_O_6_ | 3.42 | 71.0124, 59.0125, 89.0229, 85.0281, 72.9916, 113.0232, 143.8638 | D-Fructose***** |
| **12** | 0.64 | 209.0293 | - | - |  | - |
| **13** | 0.64 | 223.0452 | - | - | 129.0184, 125.0235, 205.0352, 85.0284, 72.9919, 147.0292 | - |
| **14** | 0.64 | 440.0899 | - | - |  | - |
| **15** | 0.64 | 217.0292 | - | - |  | - |
| **16** | 0.65 | 379.0820 | - | - |  | - |
| **17** | 0.65 | 404.1042 | - | - | 61.9870, 105.1312, 172.5512 | - |
| **18** | 0.65 | 341.1086 | C_12_H_22_O_11_ | 0.98 | 59.0125, 71.0124, 89.0230, 101.0230, 113.0231, 179.0552, 143.0337, 161.0439, 119.0336 | Sucrose***** |
| **19** | 0.65 | 401.1298 | - | - | 341.1087, 59.0125, 89.0230, 101.0230, 179.0555, 113.0230, 119.0335 | - |
| **20** | 0.66 | 262.0564 | - | - |  | - |
| **21** | 0.66 | 604.1724 | - | - |  | - |
| **22** | 0.66 | 539.1390 | - | - |  | - |
| **23** | 0.67 | 601.1385 | - | - | 96.9588, 78.9577 | - |
| **24** | 0.67 | 205.0349 | - | - | 125.0232, 72.9918, 99.0075, 81.0333, 85.0281, 113.0229 | - |
| **25** | 0.69 | 189.0033 | - | - |  | - |
| **26** | 0.69 | 133.0134 | C_4_H_6_O_5_ | 6.37 | 115.0023, 71.0124, 133.0129, 72.9917, 89.0230, 116.0057 | Malic acid***** |
| **27** | 0.72 | 353.0723 | - | - | 111.0078, 173.0087, 87.0076, 112.0111 | - |
| **28** | 0.72 | 435.9578 | - | - | 103.9191, 257.9837, 283.9631, 161.9615, 200.9491, 123.9454, 87.9242, 96.9590 | - |
| **29** | 0.73 | 111.0074 | - | - | 67.0177, 83.0128, 85.0284 | - |
| **30** | 0.73 | 191.0188 | - | - | 111.0074, 87.0074, 85.0281, 129.0182 | - |
| **31** | 0.74 | 290.0879 | - | - | 128.0340, 200.0556, 290.0880 | - |
| **32** | 0.74 | 586.0471 | - | - | 103.9188, 87.9239, 199.9405, 161.9610, 123.9451, 243.0983, 303.9520 | - |
| **33** | 0.76 | 128.0341 | - | - | 85.0281, 82.0286, 101.0228 | - |
| **34** | 0.77 | 341.1086 | C_12_H_22_O_11_ | 0.98 | 59.0125, 71.0124, 89.0230, 101.0230, 113.0231, 179.0551, 143.0336, 161.0437, 119.0335 | Isomer of sucrose (Jiang et al., 2006) |
| **35** | 0.78 | 377.0851 | - | - | 341.1093, 215.0321, 89.0230, 59.0125, 179.0549, 101.0229 | - |
| **36** | 1.22 | 169.0133 | C_7_H_6_O_5_ | 5.57 | 125.0231, 126.0264, 107.0128, 81.0332, 69.0332 | Isomer of gallic acid |
| **37** | 1.30 | 169.0140 | C_7_H_6_O_5_ | 1.50 | 125.0232, 126.0266, 107.0123, 81.0333, 69.0333 | Gallic acid***** |
| **38** | 1.86 | 257.1140 | C_11_H_20_N_2_O_6_ | 1.14 | 239.1031, 213.1238, 214.1273, 196.1160, 195.1131, 128.0340, 129.0374, 84.0441 | Saccharopine |
| **39** | 1.89 | 213.1242 | C_10_H_18_N_2_O_3_ | 1.25 | 170.1370, 169.1337, 126.0906, 53.0286 | Dethiobiotin |
| **40** | 2.00 | 255.0510 | C_11_H_12_O_7_ | 0.10 | 193.0499, 179.0341, 165.0547, 149.0592, 107.0490, 133.0283, 93.0334, 72.9918 | Piscidic acid |
| **41** | 2.05 | 419.1682 | C_22_H_28_O_8_ | 7.02 | 213.1240, 195.1133, 128.0341, 127.0504, 101.0231 | Rhaponticoside (Yi et al., 2007) |
| **42** | 2.27 | 153.0184 | C_7_H_6_O_4_ | 6.09 | 109.0281, 107.0124, 111.0076, 81.0332 | Protocatechuate***** |
| **43** | 2.47 | 203.0824 | C_11_H_12_N_2_O_2_ | 0.99 | 116.0492, 142.0649, 159.0913, 74.0233, 186.0545, 117.0526 | D-Tryptophan***** |
| **44** | 2.62 | 259.1297 | C_11_H_20_N_2_O_5_ | 0.95 | 241.1187, 223.1082, 197.1280, 179.1181, 130.0860, 128.0339 | Gamma-Glu-Leu |
| **45** | 2.89 | 577.1351 | C_30_H_26_O_12_ | 0.14 | 425.0880, 407.0771, 289.0717, 287.0561 | Procyanidin B (Qiu et al., 2013) |
| **46** | 3.18 | 239.0565 | - | - | 158.9243, 179.0340, 78.9577, 149.0599, 107.0492, 61.9869 | - |
| **47** | 3.18 | 186.0556 | C_11_H_9_NO_2_ | 2.43 | 142.0650, 116.0494, 143.0685, 140.0494 | 3-Indoleacrylic acid |
| **48** | 3.19 | 142.0650 | - | - | 116.0488, 59.0124, 88.6486 | - |
| **49** | 3.22 | 289.0716 | C_15_H_14_O_6_ | 0.56 | 109.0281, 123.0438, 245.0814, 203.0706, 151.0390, 137.0231, 149.0232, 187.0392, 97.0281, 205.0497 | Catechin***** |
| **50** | 3.83 | 121.0282 | - | - | 108.0204, 93.0334 | - |
| **51** | 3.92 | 313.0023 | - | - | 189.0550, 233.0451, 147.0442, 96.9587 | - |
| **52** | 4.34 | 405.1183 | C_20_H_22_O_9_ | 1.99 | 243.0659, 225.0555, 197.0600, 213.0551 | Isomer of TSG |
| **53** | 4.37 | 345.0825 | C_14_H_18_O_10_ | 0.64 | 183.0289, 182.0211, 165.0183 | Polygoacetophenoside (Qiu et al., 2013) |
| **54** | 4.43 | 421.1143 | C_20_H_22_O_10_ | -0.66 | 259.0610, 137.0232 | Pentahydroxystilbene-*O*-hexoside (Xu et al., 2009) |
| **55** | 4.47 | 301.0352 | C_15_H_10_O_7_ | 0.59 | 272.0312, 257.0452, 255.0285, 239.0339, 211.0396, 143.0493 | Delphinidin |
| **56** | 4.53 | 567.1719 | C_26_H_32_O_14_ | 0.05 | 405.1192, 243.0661, 225.0559, 173.0601, 137.0235 | Isomer of tetrahydroxystilbene-*O*-di-hexose (Thiruvengadam et al., 2014) |
| **57** | 4.69 | 189.0547 | - | - | 143.8640, 161.8747, 61.9870, 103.9189 | - |
| **58** | 4.80 | 417.1194 | C_21_H_22_O_9_ | -0.70 | 255.0660, 227.0711, 213.0549, 183.0805 | Polygonimitin B (Sun et al., 2013) |
| **59** | 4.92 | 441.0828 | C_22_H_18_O_10_ | -0.18 | 289.0717, 245.0816, 169.0133, 137.0232, 125.0232, 123.0439 | Catechin gallate (Chang et al., 2016) |
| **60** | 4.93 | 812.2493 | - | - |  | - |
| **61** | 4.94 | 439.1236 | - | - | 259.0610, 125.0231, 183.0290, 277.0721 | - |
| **62** | 4.94 | 243.0658 | C_14_H_12_O_4_ | 1.98 | 225.0550, 201.0551, 175.0754, 173.0600, 159.0441, 137.0233 | Oxyresveratrol***** |
| **63** | 4.95 | 405.1193 | C_20_H_22_O_9_ | -0.48 | 243.0659, 225.0550, 197.0598, 213.0551 | TSG***** |
| **64** | 4.95 | 495.1136 | - | - | 88.9866, 60.9917, 332.9622 | - |
| **65** | 4.95 | 468.1136 | - | - | 61.9870, 243.0659, 405.1187 | - |
| **66** | 4.95 | 847.2220 | - | - | 243.0660, 405.1190, 169.0132, 289.0716 | - |
| **67** | 4.95 | 443.0925 | - | - | 243.0657, 244.0691, 405.1184, 406.1221 | - |
| **68** | 4.95 | 441.0967 | - | - | 243.0657, 169.0132, 125.0231, 289.0717, 93.0331 | - |
| **69** | 4.98 | 269.0121 | - | - |  | - |
| **70** | 5.06 | 557.1303 | C_27_H_26_O_13_ | -0.42 | 405.1187, 313.0562, 243.0657, 169.0131, 125.0231 | Tetrahydroxystilbene-*O*-(galloyl)-glucopyranoside (Wang et al., 2005) |
| **71** | 5.19 | 417.1192 | C_21_H_22_O_9_ | -0.23 | 255.0661, 227.0709, 213.0551, 183.0806 | Isomer of polygonimitin B (Sun et al., 2013) |
| **72** | 5.37 | 447.1299 | C_22_H_24_O_10_ | -0.51 | 405.1197, 243.0662, 225.0549, 173.0599, 137.0233 | Tetrahydroxystilbene-*O*-(acetyl)-hexose (Thiruvengadam et al., 2014) |
| **73** | 5.44 | 557.1304 | C_27_H_26_O_13_ | -0.42 | 405.1180, 313.0559, 243.0657, 169.0133, 125.0231 | Isomer of tetrahydroxystilbene-*O*-(galloyl)-glucopyranoside (Wang et al., 2005) |
| **74** | 5.94 | 557.1303 | C_27_H_26_O_13_ | 0.30 | 405.1207, 313.0564, 243.0658, 169.0133, 125.0231 | Isomer of tetrahydroxystilbene-*O*-(galloyl)-glucopyranoside (Wang et al., 2005) |
| **75** | 6.02 | 393.1189 | C_19_H_22_O_9_ | 0.53 | 231.0661, 187.0758, 215.0345 | Hydroxymusizin-hexose (Liu et al., 2015) |
| **76** | 6.03 | 432.1008 | - | - |  | - |
| **77** | 6.03 | 431.0977 | C_21_H_20_O_10_ | 1.58 | 269.0453, 268.0380 | Apigenin-7-*O*-glucoside |
| **78** | 6.07 | 189.0549 | - | - | 143.8641, 161.8748, 61.9870, 174.0313 | - |
| **79** | 6.34 | 435.1293 | - | - | 231.0657, 187.0755, 145.0648, 189.0548 | - |
| **80** | 6.53 | 253.0506 | C_15_H_10_O_4_ | 0.13 | 224.0474, 209.0603, 208.0521, 197.0604, 169.0647, 135.0076 | Daidzein***** |
| **81** | 6.65 | 299.0562 | C_16_H_12_O_6_ | -0.30 | 284.0317, 256.0374, 255.0656, 240.0405, 239.0350, 228.0427, 227.0338, 211.0396 | Koparin |
| **82** | 6.67 | 511.0548 | C_21_H_20_O_13_S | 0.75 | 431.0978, 269.0453, 311.0557, 225.0551, 197.0597 | Emodin-8-*O*-*β*-D-hexose-sulfate (Krenn et al., 2003) |
| **83** | 7.21 | 407.1346 | C_20_H_24_O_9_ | 0.38 | 245.0814, 230.0577, 215.0342, 202.0627, 187.0389, 159.0437 | Torachrysone-*O*-hexose (Qiu et al., 2013) |
| **84** | 7.22 | 470.1297 | - | - | 61.9870, 245.0815, 230.0579, 215.0343 | - |
| **85** | 7.22 | 245.0816 | C_14_H_14_O_4_ | 1.36 | 230.0579, 215.0342, 159.0440, 231.0612 | Columbianetin |
| **86** | 7.23 | 408.1379 | - | - |  | - |
| **87** | 7.33 | 431.0978 | C_21_H_20_O_10_ | 0.90 | 311.0559, 269.0455, 265.0513, 241.0506, 225.0551 | Emodin-8-*O*-glucoside***** |
| **88** | 7.34 | 494.0933 | - | - | 269.0453, 431.0978, 225.0553, 240.0421 | - |
| **89** | 7.53 | 473.1088 | C_23_H_22_O_11_ | 0.29 | 269.0454, 225.0551, 240.0423 | Aloe-emodin-8-*O*-(6'-*O*-acetyl)-glucoside (Chen et al., 2001) |
| **90** | 7.54 | 517.0984 | C_24_H_22_O_13_ | 0.70 | 473.1086, 269.0453, 225.0551 | Emodin-8-*O*-(6'-*O*-carboxyacetyl)-*β*-D-glucoside (Liu et al., 2011) |
| **91** | 7.72 | 449.1451 | C_22_H_26_O_10_ | 0.49 | 389.1243, 245.0815, 230.0578, 215.0343 | Torachrysone-*O*-(acetyl)-hexose (Qiu et al., 2013) |
| **92** | 7.75 | 269.0457 | C_15_H_10_O_5_ | 0.57 | 225.0551, 241.0499, 197.0598, 105.0329, 149.0228, 164.9293 | Apigenin***** |
| **93** | 8.06 | 919.2306 | C_45_H_44_O_21_ | -0.40 | 254.0582, 458.1219, 416.1111, 225.0552, 875.2375, 713.1896, 671.1763, 255.0654, 241.0504, 213.0548 | Emodin dianthrone-hexose-(malonic acid)-hexose (Wen et al., 2012) |
| **94** | 8.15 | 285.0400 | C_15_H_10_O_6_ | 1.61 | 257.0450, 241.0495, 229.0491, 213.0546, 211.0394, 185.0589 | Kaempferol (Thiruvengadam et al., 2014) |
| **95** | 8.17 | 283.0609 | - | - | 240.0423, 212.0472 | - |
| **96** | 8.17 | 481.0906 | - | - | 283.0609, 240.0422, 268.0375 | - |
| **97** | 8.17 | 445.1137 | C_22_H_22_O_10_ | 0.72 | 283.0613, 255.0663, 199.0759 | Physcion-*O*-hexose (Qiu et al., 2013) |
| **98** | 8.19 | 431.0979 | C_21_H_20_O_10_ | 1.09 | 269.0453, 225.0551, 240.0430, 226.0586, 197.0596 | Isomer of emodin-8-*O*-glucoside |
| **99** | 8.27 | 1005.2306 | - | - | 254.0581, 458.1217, 225.0551, 713.1869, 917.2491 | - |
| **100** | 8.60 | 919.2306 | C_45_H_44_O_21_ | -0.40 | 254.0582, 458.1218, 416.1110, 225.0552, 875.2392, 713.1878, 671.1752, 255.0655, 241.0492, 213.0542 | Emodin dianthrone-hexose-(malonic acid)-hexose (Wen et al., 2012) |
| **101** | 8.63 | 283.0608 | - | - | 240.0423, 219.8447, 59.0125, 241.0448 | - |
| **102** | 8.69 | 329.2331 | C_18_H_34_O_5_ | 0.75 | 229.1442, 211.1335, 183.1382, 171.1019, 139.1119 | Aurantio-obtusin (Yu et al., 2017) |
| **103** | 8.74 | 458.1215 | - | - | 254.0581, 225.0552, 267.0662 | - |
| **104** | 8.80 | 363.0177 | - | - | 283.0612, 240.0422, 103.9189 | - |
| **105** | 9.55 | 399.1842 | - | - |  | - |
| **106** | 9.58 | 757.1769 | C_39_H_34_O_16_ | 0.67 | 713.1905, 254.0581, 458.1215, 225.0551 | Malonyl-substitution of dianthrone glycoside (Wen et al., 2012) |
| **107** | 9.76 | 757.1768 | C_39_H_34_O_16_ | 0.67 | 713.1905, 254.0581, 458.1218, 225.0551 | Isomer of malonyl-substitution of dianthrone glycoside (Wen et al., 2012) |
| **108** | 9.83 | 269.0452 | C_15_H_10_O_5_ | 1.28 | 225.0546, 241.0500 | Isomer of emodin |
| **109** | 9.83 | 349.0019 | - | - | 269.0453, 225.0551 | - |
| **110** | 9.90 | 283.0610 | C_16_H_12_O_5_ | 0.69 | 240.0423, 241.0457, 212.0473, 268.0376 | Isomer of physcion |
| **111** | 10.51 | 757.1769 | C_39_H_34_O_16_ | 0.67 | 713.1852, 254.0581, 458.1218, 225.0552 | Isomer of malonyl-substitution of dianthrone glycoside (Wen et al., 2012) |
| **112** | 10.67 | 671.1771 | C_36_H_32_O_13_ | -0.13 | 509.1249, 254.0581, 416.1110, 225.0552, 267.0659 | Emodin (10–10') emodin monosaccharide glucoside (Wen et al., 2012) |
| **113** | 10.80 | 757.1768 | C_39_H_34_O_16_ | 0.67 | 713.1887, 254.0581, 458.1217, 225.0550 | Isomer of malonyl-substitution of dianthrone glycoside (Wen et al., 2012) |
| **114** | 11.16 | 311.0564 | C_17_H_12_O_6_ | -0.93 | 283.0612, 269.0462, 268.0374, 255.0663, 240.0427, 224.0478, 212.0472, 196.0526, 184.0519 | Acetyl-aloe-emodin (Li et al., 2013) |
| **115** | 11.74 | 473.2175 | - | - | 59.0127 | - |
| **116** | 11.81 | 368.9702 | - | - | 322.9649, 269.0451, 350.9593, 240.0413, 99.9245 | - |
| **117** | 11.82 | 270.0483 | - | - |  | - |
| **118** | 11.82 | 431.8842 | - | - |  | - |
| **119** | 11.82 | 429.8863 | - | - | 160.8412, 393.9103 | - |
| **120** | 11.82 | 269.0454 | C_15_H_10_O_5_ | 0.92 | 225.0551, 241.0500, 197.0599, 169.0650, 181.0649 | Emodin***** |
| **121** | 12.77 | 285.0402 | C_15_H_10_O_6_ | 0.91 | 257.0449, 241.0498, 213.0538 | Isomer of kaempferol (Thiruvengadam et al., 2014) |
| **122** | 13.14 | 233.1540 | - | - | 59.0125, 188.9846 | - |
| **123** | 13.21 | 509.1237 | C_30_H_22_O_8_ | 0.96 | 254.0583, 226.0619 | Aloeemodin dianthrone (Yang et al., 2005) |
| **124** | 13.83 | 509.1237 | C_30_H_22_O_8_ | 0.96 | 254.0581, 226.0627 | Isomer of aloeemodin dianthrone (Yang et al., 2005) |
| **125** | 13.88 | 233.1540 | - | - | 59.0125, 188.9848 | - |
| **126** | 14.36 | 283.0610 | C_16_H_12_O_5_ | 0.69 | 240.0423, 241.0457, 212.0471, 184.0520, 268.0373 | Physcion***** |
| **127** | 15.11 | 433.2356 | - | - | 152.9948, 78.9577, 96.9682 | - |
| **128** | 15.17 | 523.1393 | C_31_H_24_O_8_ | 1.07 | 254.0582 | Emodin (10/10') physcion dianthrone glycoside (Wen et al., 2012) |
| **129** | 15.46 | 523.1393 | C_31_H_24_O_8_ | 1.07 | 254.0580 | Isomer of emodin (10/10') physcion dianthrone glycoside (Wen et al., 2012) |
| **130** | 16.91 | 277.2173 | C_18_H_32_O_2_ | 0.01 | 243.2108, 191.1443, 177.1278, 163.1111, 149.0231, 137.1323, 121.1013, 109.1015, 107.0860, 95.0855, 83.0859, 55.0551 | 9*Z*-Octadecen-12-ynoicacid (Xu et al., 2012) |
| **131** | 17.23 | 227.2015 | C_14_H_27_O_2_ | 1.76 | 180.8305 | Tetradecanoic acid (Xu et al., 2012) |
| **132** | 17.50 | 381.2808 | - | - | 337.2183, 163.1122, 338.2223 | - |
| **133** | 17.88 | 695.3114 | - | - | 59.0127, 78.9579, 279.2331, 152.9949 | - |
| **134** | 18.01 | 279.2326 | C_18_H_32_O_2_ | 1.27 | 261.2213, 59.0124 | Linoleic acid***** |
| **135** | 19.04 | 255.2329 | C_16_H_31_O_2_ | 0.21 | 219.8447 | *n*-Hexadecanoic acid (Xu et al., 2012) |
| **136** | 19.24 | 281.2484 | C_18_H_33_O_2_ | 0.72 | 243.7902, 136.8905 | 9-Octadecadienoic acid (9*Z*) (Xu et al., 2012) |

*: means the compounds were compared with standards.


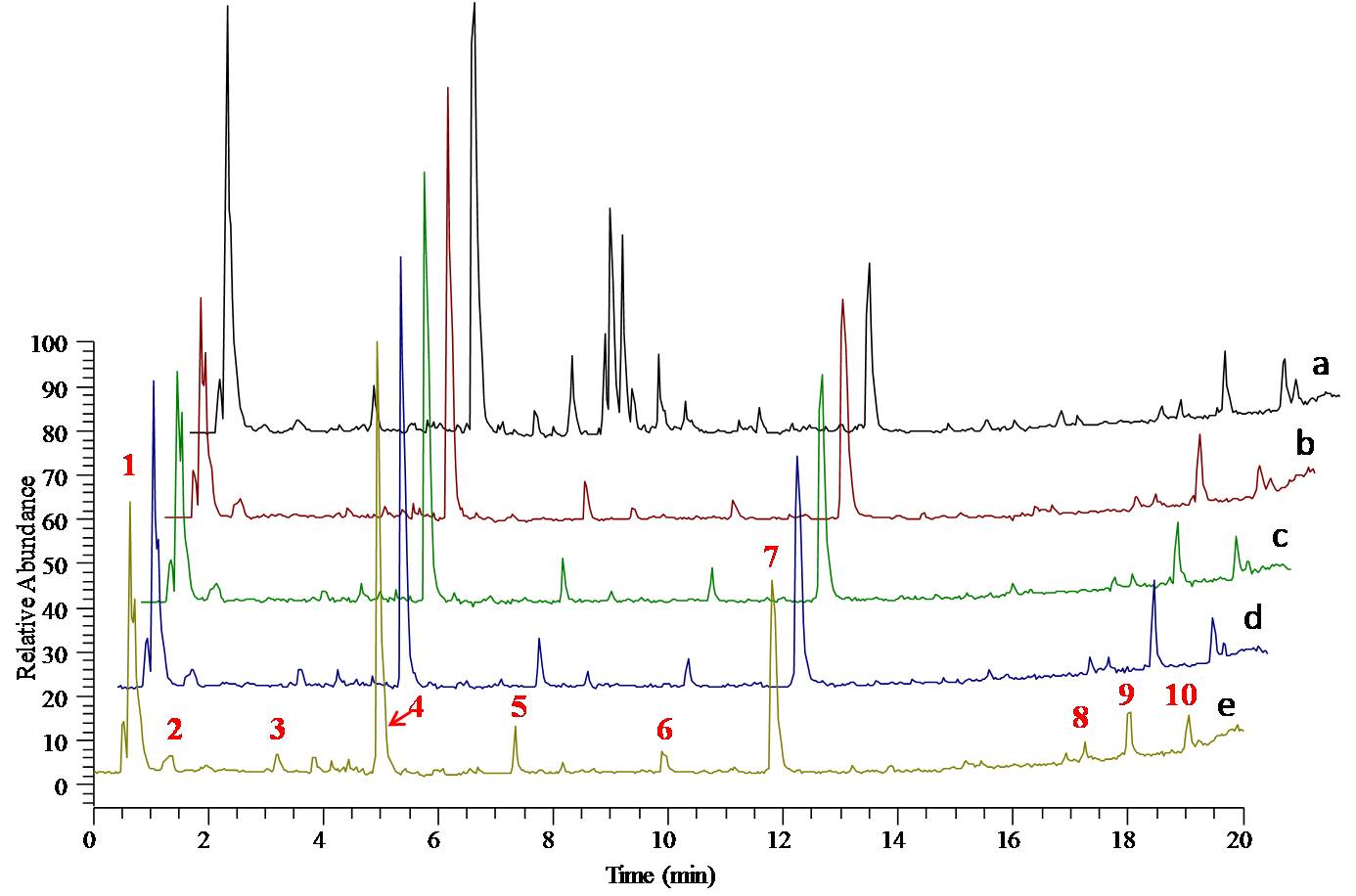


**FIGURE. S1.** Typical TIC chromatograms of R-PM and four kinds of P-PM.

(a: R-PM; b: W-PM; c: YRW-PM; d: BD-PM; e: BD+YRW-PM)


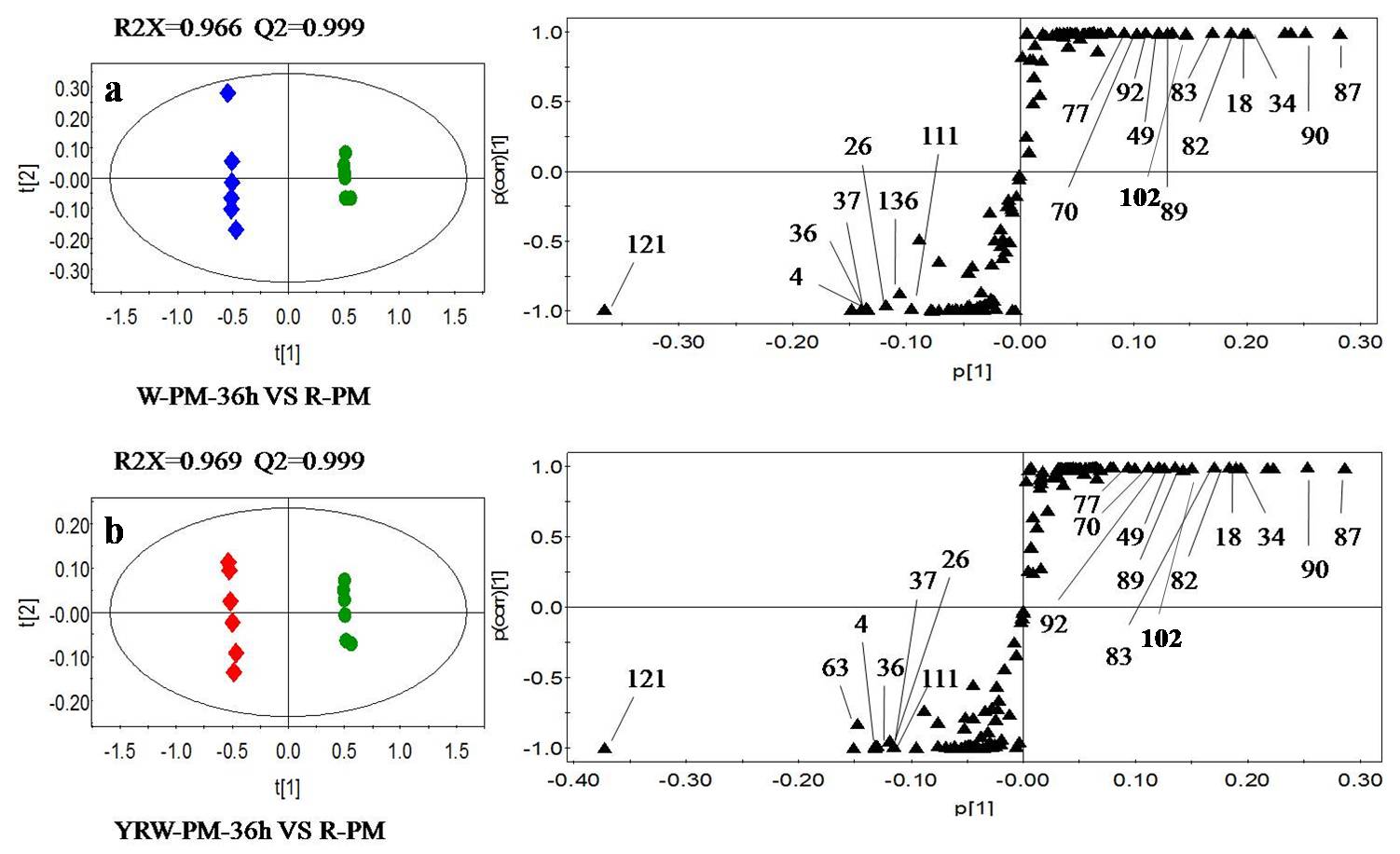


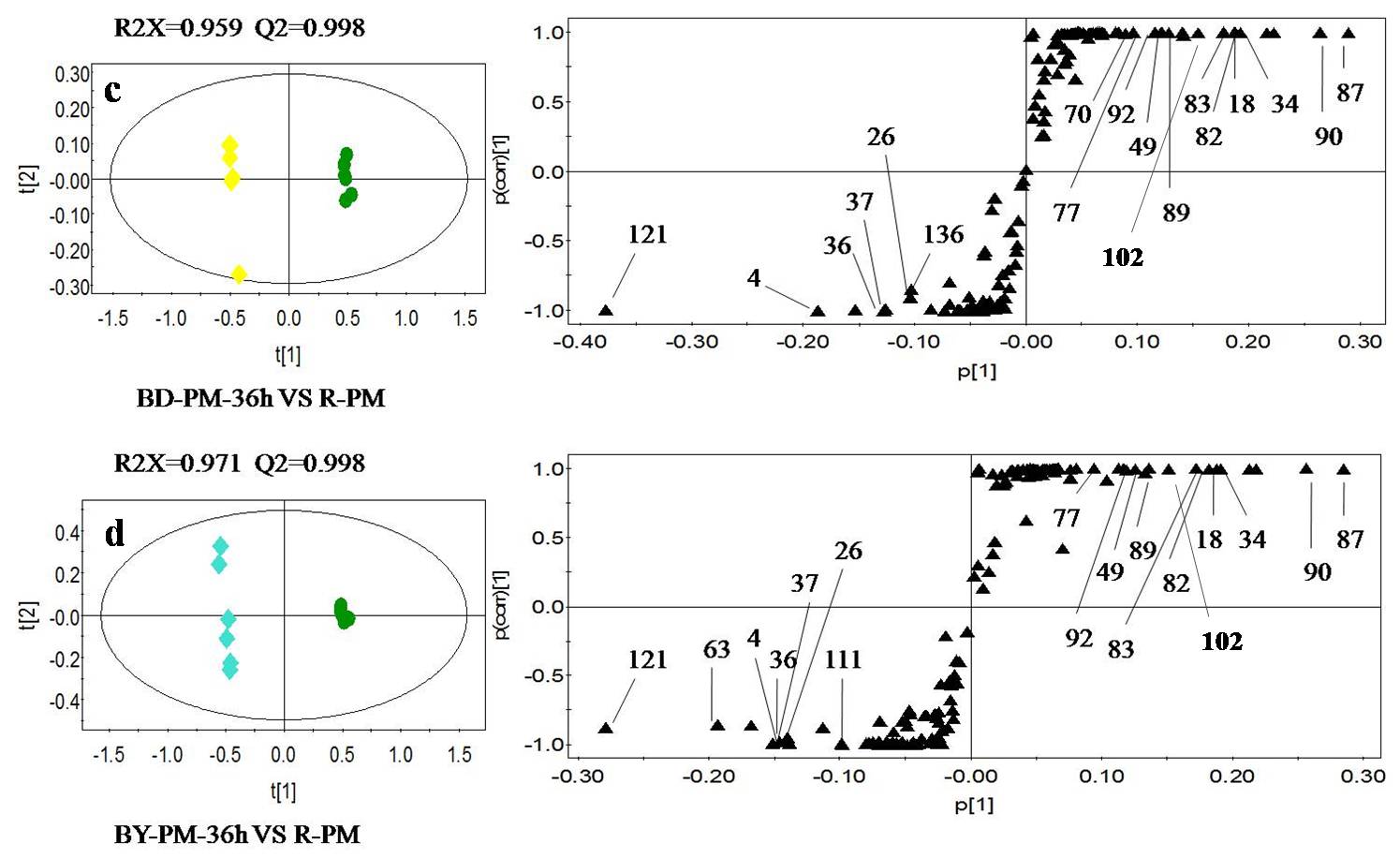


**FIGURE S2.** OPLS-DA scores plots (left) and S-plots (right) showing the metabolic differences between four kinds of P-PM and R-PM for (a) water-PM ( ) vs R-PM ( ), (b) YRM-PM ( ) vs R-PM ( ), (c) BD- PM ( ) vs R-PM ( ), (d) BY-PM ( ) vs R-PM ( ) at 36 h.

(The numbers of metabolites in S-plot were identical with those in Table S1)


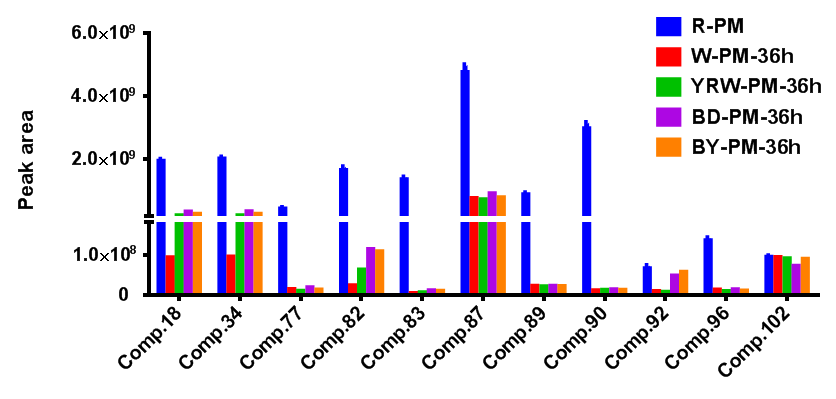


**FIGURE S3.** The abundance map of the preferred markers from OPLS-DA annlysis among R-PM and P-PM at 36 h
